# PREVALENCE OF ***HELICOBACTER PYLORI*** TEN YEARS AGO COMPARED TO THE CURRENT PREVALENCE IN PATIENTS UNDERGOING UPPER ENDOSCOPY

**DOI:** 10.1590/0102-6720201600030006

**Published:** 2016

**Authors:** Sandra FRUGIS, Nicolau Gregori CZECZKO, Osvaldo MALAFAIA, Artur Adolfo PARADA, Paula Bechara POLETTI, Thiago Festa SECCHI, Matheus DEGIOVANI, Alécio RAMPANAZZO-NETO, Mariza D. D´AGOSTINO

**Affiliations:** 1 Postgraduate Program in Principles of Surgery, Evangelic Faculty of Paraná/University Evangelic Hospital of Curitiba/Medical Research Institute, Curitiba, PR, Brazil; 2 Gastrointestinal Endoscopy Service, 9 of July Hospital, São Paulo, SP, Brazil

**Keywords:** Helicobacter pylori, Endoscopy, Urease test

## Abstract

**Background:**

Helicobacter pylori has been extensively studied since 1982 it is estimated that 50% of the world population is affected. The literature lacks studies that show the change of its prevalence in the same population over time.

**Aim:**

To compare the prevalence of H. pylori in 10 years interval in a population that was submitted to upper endoscopy in the same endoscopy service.

**Method:**

Observational, retrospective and cross-sectional study comparing the prevalence of H. pylori in two samples with 10 years apart (2004 and 2014) who underwent endoscopy with biopsy and urease. Patients were studied in three consecutive months of 2004, compared to three consecutive months of 2014. The total number of patients was 2536, and 1406 in 2004 and 1130 in 2014.

**Results:**

There were positive for H. pylori in 17 % of the sample as a whole. There was a significant decrease in the prevalence from 19.3% in 2004 to 14.1% in 2014 (p<0.005).

**Conclusion:**

There was a 5.2% reduction in the prevalence of H. pylori comparing two periods of three consecutive months with 10 years apart in two equivalent population samples.

## RESUMO

**Racional:**

Helicobacter pylori vem sendo amplamente estudado desde 1982 estimando-se que 50% da população mundial esteja afetada. A literatura carece de estudos que mostrem a mudança de sua prevalência em uma mesma população ao longo do tempo.

**Objetivo:**

Comparar a prevalência do H.pylori no intervalo de 10 anos em população que realizou endoscopia digestiva alta no mesmo serviço de endoscopia.

**Método:**

Estudo observacional, retrospectivo e transversal, comparando a prevalência de H. pylori em duas amostras com intervalo de 10 anos (2004 e 2014) que realizaram endoscopia digestiva alta com biópsias e teste da urease para a pesquisa de H. pylori. Foram estudados pacientes em três meses consecutivos de 2004, comparados aos de três meses consecutivos de 2014. O número total de pacientes avaliados foi 2536, sendo 1406 em 2004 e 1130 em 2014.

**Resultados:**

Constatou-se resultado positivo para H.pylori em 17% da amostra como um todo. Houve queda significativa da prevalência de H.pylori de 19,3% em 2004 para 14,1% em 2014 (p<0.005).

**Conclusão:**

Houve redução de 5,2% da prevalência de H. pylori comparando-se dois períodos de três meses consecutivos com intervalo de 10 anos em duas amostras populacionais equivalentes.

## INTRODUCTION

Gastric micro-organisms were observed for more than 100 years^7^, but its association with gastric diseases was recognized from 1982, when Marshall and Warren identified and subjected to culture the bacteria *Campylobacter pyloridis* later reclassified as *Helycobacter pylori* (HP)^7^ ^,^ ^21^.

This gram-negative bacterium, a spiral, microaerophilic aspect, quite resistant and may remain alive for long periods of time outside the human body, water, vegetable and feces. Contamination of water supply reservoirs in developing countries can serve as an environmental source. Using the polymerase chain reaction technique, HP was found in most water samples from supply reservoirs in endemic infected areas^7^. In a study conducted in South Korea, analyzing risk factors for HP, there was contamination of 3% natural source of water, 92% in the city reservoirs and 66% in bottled water^20^.

HP has been diagnosed throughout the world and in all age groups. It is estimated that 50% of the world population is infected^7^. It was isolated in animals, milk, raw vegetables, feces, vomiting and water. Its transmission is oral-oral and fecal-oral between humans, with a higher prevalence in the low-income population, where contamination begins during childhood linked with poor housing, food and hygiene^1^ ^,^ ^12^ ^,^ ^20^ ^,^ ^27^ ^,^ ^29^. In developing countries, where most children are infected before age 10, the adult prevalence reaches 80% before 50 years^7^. Several studies indicate that decrease in incidence and prevalence of HP progressively in the last 20 years is related to the industrialization and improvement of health and socio-cultural conditions in different countries ^1^ ^,^ ^16^ ^,^ ^20^ ^,^ ^24^ ^,^ ^27^ ^,^ ^28^ ^,^ ^29^.

This bacterium is biochemically characterized as being urease-dependent, being of importance to invasive and noninvasive diagnostic tests^9^ ^,^ ^15^ ^,^ ^18^ ^,^ ^19^ ^,^ ^22^ ^,^ ^24^ ^,^ ^25^ ^,^ ^27^ ^,^ ^28^ ^,^ ^29^.

The recommendation of the American College of Gastroenterology in 2007 is to realize HP urease test in patients with dyspepsia, active ulcer or history of ulcer, lymphomas, in chronic use of proton pump inhibitors and steroid anti-inflammatories^3^; in anemic patients with vitamin B12 and iron deficiency; and asymptomatic patients with a family history of gastric cancer or in families with HP treatment^4^ ^,^ ^9^ ^,^ ^10^ ^,^ ^23^ ^,^ ^24^ ^,^ ^29^. In Brazil, the III HP Brazilian Consensus in 2012, had as one of the goals disclose and guide dietetic and hygiene measures to the population, develop strategies with the Ministry of Health to improve sanitation and water reservoirs and guide health professionals regarding HP prevention , diagnosis and treatment^2^ ^,^ ^3^ ^,^ ^4^ ^,^ ^6^ ^,^ ^9^ ^,^ ^11^ ^,^ ^12^ ^,^ ^14^ ^,^ ^25^ ^,^ ^30^.

In São Paulo, SP, Brazil, the total estimated population in 2015 was 11,968,000 people. The study of HP prevalence in two periods with an interval of 10 years, in a population with similar characteristics, can provide information on how this infection is behaving over time and help to orientate prevention and treatment. It is likely that, as in other countries, can be detected changes in prevalence in accordance with the dietetic and hygiene changing conditions of each region^7^ ^,^ ^20^ ^,^ ^29^.

Thus, this epidemiological study aims to compare the prevalence of HP in the 10 year range in population that held endoscopy in the same endoscopy service and with the same population characteristics.

## METHOD

This is an observational, retrospective and cross study comparing the prevalence of HP in two groups of patients, 10 years apart (2004 and 2014) who underwent endoscopy with biopsy and urease test for HP systematically done in the Service of Digestive Endoscopy, Diagnostic Center and Endoscopic Therapy of São Paulo, 9 of July Hospital, São Paulo, SP, Brazil.

The total number of patients who underwent endoscopy in 2004 was 6121 patients, and in 2014 to 6352. Were assessed those who underwent tests in three consecutive months (January, February and March) of 2004 (n=1406) and 2014 (n=1130), a total of 2536 patients. Were excluded patients on anticoagulant treatment and unable to perform biopsies. All endoscopies were with sedation by midazolam and fentanyl citrate.

Systematically were done three biopsies (antrum, incisura angularis and gastric body) and urease test in all endoscopies. The following variables were analyzed: year, gender, HP test results, age, gross appearance of the mucosa (normal endoscopy or pathological changes).

### Statistical analysis

The data were submitted to the Pearson chi-square test.

## RESULTS

The collected data are summarized in Table 1.

**TABLE 1:** Descriptive data analysis

Variables	Total number of patients n=2536(100%)	Patients HP+ n=432(100%)	Patients HP- n=2104(100%)
Year			
Total 2004	1406 (100%)	272 (19.3%)	1134 (80.7%)
Total 2014	1130 (100%)	160 (14.1%)	970 (85.9%)
Gender			
Total female	1624 (100%)	256 (15.7%)	1368 (84.3%)
Total male	912 (100%)	176 (19.3%)	736 (80.7%)
HP resultsTotal sample			
	2536 (100%)	432(17.0%)	2104(83.0%)
Age			
< 10 y	2536 (100%)	432 (100%)	2104 (100%)
	09 (0.4%)	01 (0.2%)	08 (0.4%)
11-20 y	69 (2.7%)	04 (1.0%)	65 (3.1%)
21-30 y	377 (14.8%)	61 (14.2%)	316(15.0%)
31-40 y	526 (20.7%)	80 (18.5%)	446(21.2%)
41-50 y	484 (19.2%)	190(43.8%)	294(14.0%)
51-60 y	534 (21.0%)	45 (10.4%)	489(23.3%)
Over 61 y	537 (21.2%)	51 (11.9%)	486(23.0%)
Normal endoscopy/with alterations			
Total normal endoscopies	134 (100%)	22 (16.5%)	112 (83.5%)
Total endoscopies with alterations	2402 (100%)	410 (17.0%)	1992 (83.0%)

In 2004 the prevalence of HP was equal to 19.3%; already in 2014 it dropped down to 14.1%.

The proportion of women in total (64%) was higher than that of men (36%). Of the 1624 women, 256 (15.7%) were HP+ and 1368 (84.3%) HP-. Of the 912 men, 176 (19.3%) were HP+ and 736 (80.7%) HP-.

In the sample as a whole (n=2536) there were 17% (n=432) positive results for HP. In 2004 its prevalence was 19.3% (n=272), and in 2014 dropped to 14.1% (n=160), p<0.005 (Table 2).

**TABLE 2:**
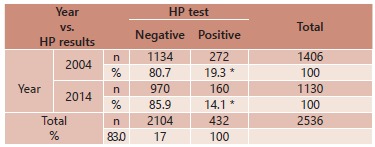
HP result vs. year

*p<0,005; HP=*Helicobacter pylori*

**FIGURE 1:**
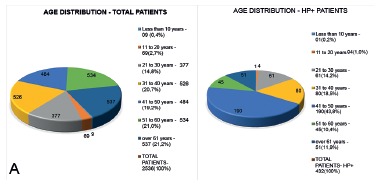
Age distribution vs. presence of HP

In Figure 1A it can be observed the distribution of all patients (n=2536) by age group. In Figure 1B it can be seen the distribution only HP+ (n=432, 17% of the total sample) distributed for the same age groups.

In 2004 from 1406 patients, HP- was seen in 1134 patients, being 51 with normal endoscopy (4.5%) and 1083 showing some alteration (95.5%). Of the 272 patients with PH+ seven had normal endoscopy (2.6%) and 265 related some disease (97.4%). The total of normal endoscopies in 2004 was in 58 patients (Table 3).

**TABLE 3:**
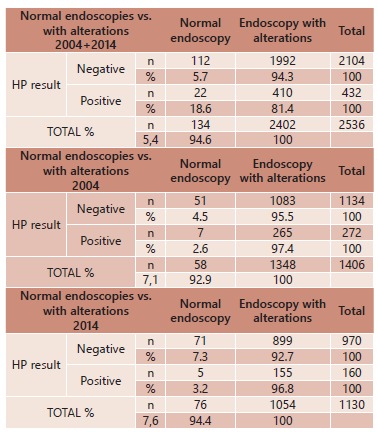
Normal endoscopy, with alterations and HP+/- in 2004+2014, 2004 e 2014

In 2014 from 1130 patients HP- was seen in 970, being 71 with normal endoscopy (7.3%) and 899 with alterations (92.7%). Of the 160 HP+ five had normal endoscopy (3.2%) and 155 with some disease (96.8%). The total of normal endoscopies in 2014 was 76 (Table 3).

There was an increase of normal and HP- endoscopies in 10-year period (4.5% in 2004 and 7.3% in 2014). There was a significant reduction of HP+ in patients with endoscopy with some disease (97.4% in 2004 and 96.8% in 2014) in the same period (Table 3). There was a reduction of 5.2% of HP+ prevalence (19.3% in 2004 to 14.1% in 2014) that was statistically significant (p<0.005). The sample consisted mostly of women (64%). The highest incidence of HP+ in this sample was between 41 and 50 years (43.87%).

**FIGURE 2:**
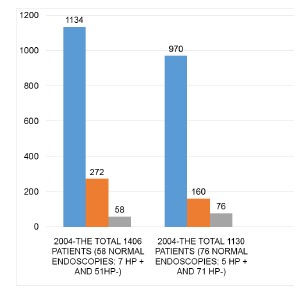
Distribution of patients HP+, HP- and normals in 2004 and 2014

Of the total of 2536 endoscopes in both years, only 134 were normal with no mucosal alterations (Figure 2).

## DISCUSSION

The variation of HP prevalence in different regions of the world is mainly related to socioeconomic factors^7^ ^,^ ^16^ ^,^ ^20^ ^,^ ^25^ ^,^ ^29^. It is observed that in developed countries it is lower since childhood, and the regions that showed socioeconomic growth in the past 20 years decreased significantly^20^ ^,^ ^29^.

In developing countries, most children are infected before age 10 and the adult prevalence reaches 80% before age 50; in developed countries like the United States, the incidence of HP is rare in children under 10 years and increases to 10% between 18-30 years^7^ ^,^ ^29^.

This is attributed to the fact that in developed societies, the population has access to sanitation, hygiene, health (medical and dental) and guidance for prevention and treatment^11,12,14,17,18,19,26,29 30^.

Because HP is the most common infection in humans, and as a consequence of its chronic character, may trigger serious diseases with considerable morbidity^1^ ^,^ ^3^ ^,^ ^4^ ^,^ ^5^ ^,^ ^8^ ^,^ ^13^ ^,^ ^19^ ^,^ ^20^ ^,^ ^30^. Comparative study of its prevalence in distinct regions over the years is of great importance to analyze if the health improvement of population translate into reducing the prevalence, as well as guiding strategies and guidelines for the prevention and specific treatment for each area proportional to the degree of socioeconomic and cultural development ^2^ ^,^ ^7^ ^,^ ^11^ ^,^ ^12^ ^,^ ^14^ ^,^ ^20^ ^,^ ^26^ ^,^ ^28^ ^,^ ^29^.

Regarding the studied São Paulo population in private service, the prevalence of HP fell significantly in a decade, and there was also a reduction in its incidence in patients with endoscopies with some disease.

It is possible that these findings are a result of greater public awareness of the quality of food, the preventive health care (medical and dental) and discipline following the medical guidelines. It is possible also to believe that an important factor for this favorable development is broad access to information that has been occurring in recent years. If these conditions remain unchanged over time, will happen further decline in this prevalence, as currently already occurs in developed countries.

Further studies are needed in regions with different levels of socioeconomic development, to check the HP prevalence in the population in the coming years.

## CONCLUSION

It was observed significant reduction in the prevalence of HP comparing two periods of three consecutive months with an interval of 10 years, in two similar population samples. There was a reduction of 5.2% of HP+ prevalence, falling from 19.3% in 2004 to 14.1% in 2014.
